# Identification of Mispairing Omic Signatures in Chinese Hamster Ovary (CHO) Cells Producing a Tri-Specific Antibody

**DOI:** 10.3390/biomedicines11112890

**Published:** 2023-10-25

**Authors:** Maria João Sebastião, Michael Hoffman, José Escandell, Fatemeh Tousi, Jin Zhang, Bruno Figueroa, Christine DeMaria, Patrícia Gomes-Alves

**Affiliations:** 1 iBET, Instituto de Biologia Experimental e Tecnológica, Apartado 12, 2780-901 Oeiras, Portugal; msebastiao@ibet.pt (M.J.S.);; 2 ITQB-NOVA, Instituto de Tecnologia Química e Biológica António Xavier, Universidade Nova de Lisboa, Av. da República, 2780-157 Oeiras, Portugal; 3 Sanofi Cell Line and Cell Bank Development, Mammalian Platform, Global CMC Development, Framingham, MA 01701, USAbruno.figueroa@sanofi.com (B.F.);; 4 Sanofi Bioanalytics Development, Global CMC Development, Framingham, MA 01701, USA

**Keywords:** multispecific antibodies, mispairing, transcriptomics, proteomics, CHO cells

## Abstract

Monoclonal antibody-based therapy has shown efficacy against cancer, autoimmune, infectious, and inflammatory diseases. Multispecific antibodies (MsAbs), including trispecifics (tsAbs), offer enhanced therapeutic potential by targeting different epitopes. However, when co-expressed from three or more different polypeptide chains, MsAb production can lead to incorrect chain assembly and co-production of mispaired species with impaired biological activity. Moreover, mispairing carries significant challenges for downstream purification, decreasing yields and increasing the cost of bioprocess development. In this study, quantitative transcriptomics and proteomics analyses were employed to investigate which signaling pathways correlated with low and high mispairing clone signatures. Gene and protein expression profiles of Chinese hamster ovary (CHO) clones producing an tsAb were analyzed in the exponential growth and stationary (tsAb production) phase of fed-batch culture. Functional analysis revealed activated endoplasmic reticulum stress in high mispairing clones in both culture phases, while low mispairing clones exhibited expression profiles indicative of activated protein translation, as well as higher endocytosis and target protein degradation, suggesting the clearance of unfolded proteins through ubiquitin-mediated mechanisms. In addition, through transcriptomic profiling, we identified a group of genes that have the potential to be used as a biomarker panel tool for identifying high mispairing levels in the early stages of bioprocess development.

## 1. Introduction

Since the discovery of hybridoma technology [1], monoclonal antibodies have greatly advanced, becoming a pivotal and powerful tool for knowledge-driven scientific research and clinical applications. Multispecific antibodies (MsAbs) contain binding sites to different epitopes and can therefore bind more than one type of target antigen simultaneously. MsAbs are an emerging therapeutic modality, better equipped to deal with diseases with complex pathogenesis, where the activation or repression of a single target mediator is insufficient to produce the desired phenotypic outcome. In addition, this multitarget approach avoids the higher costs and regulatory hurdles associated with the production and approval of several monospecific antibodies against different targets.

MsAbs have already been applied for different purposes, including simultaneous binding of different epitopes on the same receptor [2], promotion of enzyme/substrate co-localization [3,4], and targeted T-cell mediated cytotoxicity through simultaneously activating T-cells and the targeting of tumor antigens [5,6,7]. Promising examples include the development of several types of trispecific antibody (tsAb) T-cell engagers, simultaneously activating T-cells receptors, while binding to receptors specific for cancer cells [8,9]. Another example constitutes the use of this class of antibodies to interact with three different HIV-1 envelope sites, exhibiting higher potency than the previously developed neutralizing single epitope mAbs and conferring immunity in a non-human primate model [10].

According to clinicaltrials.gov (accessed on 22 August 2023) records, there are over 227 ongoing studies in clinical development using MsAbs to target cancer, autoimmune, inflammatory, and other diseases (criteria: recruiting, not yet recruiting, active not recruiting, enrolling by invitation, and approved for marketing; terms “bispecific antibody”, “trispecific antibody” and/or “multispecific antibody”). So far, five MsAbs have been approved for clinical use: catumaxomab (Removab^®^, discontinued) [11], blinatumomab (Blincyto^®^) [6,12], Emicizumab (Hemlibra^®^) [4], amivantamab (Rybrevantand^®^) [13], and faricimab (Vabysmo™) [14]. Besides therapeutic applications, MsAbs have also been explored for detection and diagnosis strategies [15,16,17].

Production of many MsAb formats requires the co-expression of more than two different polypeptide chains, which can result in incorrect chain pairing. This leads to the production of undesired mispaired species (such as heavy chain homodimerization and non-cognate assembly of heavy and light chains), negatively affecting molecule stability and antigen binding capability [18,19]. 

Several strategies have been applied to avoid or decrease mispaired MsAb species through heavy and light chain domain engineering (reviewed in [18,20]), such as the knobs-into-holes design that favors heterodimeric assembly of heavy chains using CH3 domain engineering [21], engineering of VH/VL domain light chain interfaces [22], and automated in silico platforms to screen different MsAb formats [23]. Even with the significant improvements made through these types of sequence-dependent strategies, some degree of mispairing is still observed, impacting product quality and reducing the yield of the correct form of MsAb. Mispaired MsAb species are therefore considered product related impurities, often presenting differences in molecular mass, net charge, hydrophobicity, and hydrodynamic diameter, as well as a higher tendency to form protein aggregates, posing considerable challenges for characterization analysis and downstream purification steps. Whereas strategies using different chromatographic, electrophoretic, and mass spectrometry (MS)-based methods have been explored to streamline the analysis and purification of MsAbs [18,19,24,25], minimizing MsAb-mispairing-related impurities should also be addressed in earlier stages of bioprocess development, in order to mitigate the burden on analytical and purification steps, which are a major contributor to development costs and longer timelines.

Mispairing levels are dependent not only on the specificities of each different MsAb molecule, but also on the producer cell line. Although cell clone screening is often performed early in bioprocess development, to select clones with a more favorable growth and product titer profile, screening for clones that yield low levels of mispaired antibody products is commonly performed only in initial uncloned pool populations or mid-late stages of clone development, due to the high costs and timelines of mispairing analytical characterization.

Here, aiming at defining which intrinsic cell molecular profiles correlate with improved MsAb mispairing levels, we investigated how gene and protein expression profiles correlate with MsAb mispairing level in CHO clones producing an tsAb.

A quantitative transcriptomics and proteomics analysis was applied to CHO clones producing tsAb with different mispairing levels in both exponential growth (day 5) and stationary/tsAb production (day 10) phases in a fed-batch culture. Functional assessment of gene and protein expression fingerprints revealed activated endoplasmic reticulum stress in high mispairing clones, while low mispairing clone analysis pointed towards activated protein translation levels, as well as higher endocytosis and ubiquitin-mediated protein degradation. The transcriptomic profiles obtained were also screened for potential biomarker genes that could be easily screened using reverse transcriptase (RT) quantitative polymerase chain reaction (qPCR), and used as a high throughput methodology to identify candidate low mispairing clones in early stages of cell line development, decreasing costs and timelines for MsAb production.

## 2. Materials and Methods

Cell culture: CHO DXB11 host-cell-derived clones producing a trispecific antibody (tsAb) were cultivated under a proprietary 12-day stirred tank fed batch culture process in 15 mL micro-bioreactors from a 48-vessel ambr™ system (Sartorius, Göttingen, Germany). Three independent ambr™ runs were performed. In the results displayed, duplicate or triplicate vessels for each clone were included. To ensure consistency between clones, the same process parameters were applied, including initial cell density (1 × 10^6^ viable cells/mL), temperature (36.5 °C), feeding regime, oxygen (40% DO), and pH control (target pH 7). During culture, the viable cell density (VCD) and viability were determined using a Vi-Cell XR device (Beckman Coulter, Brea, CA, USA). Analysis of total IgG, ammonia, lactate, and glucose throughout the culture was performed using a CedexBio instrument (Roche, Basel, Switzerland) for feeding strategy purposes. At day 12 of culture, titer was determined using an Octet Red96 Instrument with Protein A biosensors (ForteBio, Fremont, CA, USA). Clarified cell culture harvest and cell pellets were stored at −80 °C until further purification and/or analysis.

Differential gene expression analysis: Total RNA was extracted using an RNeasy Mini Kit (Qiagen, Venlo, The Netherlands), according to the manufacturer’s instructions, and quantified using Lunatic equipment (Unchained Labs, Pleasanton, CA, USA) through absorbance at 260 nm with background correction at 340 nm. Purity of RNA was analyzed using a 260/280 nm and 260/230 nm absorbance ratio and through capillary electrophoresis using a fragment analyzer system (Agilent, Santa Clara, CA, USA). RNA-seq libraries were prepared using the QuantSeq 3′ mRNA-Seq Library Prep Kit (FWD) (Lexogen, Vienna, Austria) according to the manufacturer’s instructions. The RNA-Seq libraries were sequenced using the Illumina NextSeq500 (Illumina, San Diego, CA, USA), to produce 75 bp single-end reads for each sample. Quality control, library preparation, and sequencing was performed at the Genomics Unit in the Instituto Gulbenkian de Ciência (Oeiras, Portugal). Raw sequences were mapped to the Ensembl Mouse Genome Assembly GRCm38:CM001002.2. Mapping, trimming, alignment, and gene count were performed using BlueBee platform (https://www.bluebee.com/, accessed on 27 December 2022) using *FastQ Merging 1.2.0* and *FWD-UMI CHO-K1 Lexogen QuantSeq 2.2.3* pipelines. The full list of transcripts can be accessed in Supplementary File S1. 

Validation of Differential gene expression by RT-qPCR: Ten genes were selected for validation of expression using qPCR (*Ntn1*, *Fcho1*, *Eps*, *Rpl28*, *Fbxl20*, *Bicd1p*, *Ccl2*, *Grhl2*, *S100a16*, and *Vasn*). Selection of genes was based on differential expression in low- vs. high-mispairing clones. Total RNA was extracted using an RNeasy Mini Kit (Qiagen, Venlo, The Netherlands), according to the manufacturer’s instructions. Reverse transcription of total RNA was performed using a transcriptor high fidelity cDNA synthesis kit (Roche, Basel, Switzerland) kit, according to the manufacturer’s instructions. Gene expression was quantified with SYBR Green (SYBR Green I Master mix, Roche, Basel, Switzerland) on LightCycler 480 (LC480, Roche, Basel, Switzerland) equipment using gene-specific primers. All experiments were performed in triplicate. mRNA transcripts were normalized to β-actin. The sequences of the primers used are depicted in Table 1.

Differential protein expression analysis:

Protein Extraction and Digestion: Proteins were extracted, quantified, and processed from cell pellets, as described elsewhere [26]. Briefly, cell pellets were resuspended in lysis buffer (50 mM Tris (pH 7.8); 250 mM Sucrose; 2 mM EDTA) with protease inhibitors and incubated on ice for 10 min. Cells were lysed with 30 passes through 301/2 Gauge needles at 4 °C. The cell debris, unbroken nuclei, and other membrane proteins were pelleted and removed through centrifugation at 1000× *g* for 10 min at 4 °C, and the total protein amount in the supernatant was quantified using a Microplate BCA Protein Assay Kit (Thermo Scientific, Waltham, MA, USA). 

Proteomics workflow was performed as described previously [27], including protein digestion, generation of the spectral reference library, and SWATH-MS analysis. 

Briefly, proteins were subjected to gel electrophoresis and digested with trypsin. For the generation of the spectral reference library, each sample (2.5 μg) was used for information-dependent acquisition (IDA) analysis with NanoLC–MS using a TripleTOF 6600 (ABSciex, Framingham, MA, USA). The spectral library was created by combining all IDA raw files using ProteinPilot software (v5.0 ABSciex). For SWATH-MS quantitative analysis, 2.5 μg of each sample was subjected to 3 SWATH runs.

A total of 714 proteins were quantified under these conditions. The full list of quantified proteins can be accessed in Supplementary File S2. 

Proteome and Transcriptome statistical and functional analysis: To identify differentially expressed proteins and genes between low and high mispairing clones (low mispairing if reported correct tsAb form ≥ 90%; high mispairing if correct tsAb form < 90%), quantitative data from SWATH and RNAseq 3′sequencing were analyzed.

Statistical analysis of proteome SWATH data was performed on logarithmized intensities for protein normalized peak areas. Differential expression of proteins was identified by performing *t*-test using the Perseus software environment version 2.0.11 [28]. Statistical analysis of transcriptome gene counts was performed using DESeq2 R package version 1.36.0, including gene count normalization, independent filtering, and *t*-test analysis [29]. Only genes with baseMean values equal to or higher than 1 were analyzed. 

Resulting *p*-values and fold changes were used to define up- and downregulated proteins/genes. Differentially expressed molecules were defined as those which showed a fold change greater than 1.5 (upregulated) or lower than 0.67 (downregulated) and *p*-values lower than 0.05. Principal component analysis mapping was performed using Perseus software environment [28]. Pathway analysis was performed using Ingenuity Pathway Analysis software (IPA, Qiagen, Hilden, Germany), by uploading the quantified molecule list (only molecules with *p*-value < 0.05) and respective fold change. Statistically significant representation of biological functions and canonical pathways was identified based on IPA *p*-value, displayed as −log (*p*-value). This probability score is calculated by taking into account the total number of molecules known to be associated with a given function or pathway and their representation in the experimental dataset. Prediction of inhibition and activation of biological functions, canonical pathways, and upstream regulators was based on IPA z-score, a statistical measure of the match between the expected relationship direction and observed molecule expression resulting in activation (z-score ≥ 2) or inhibition (z-score ≤ −2) of the respective pathway. 

## 3. Results

CHO clones were cultivated in a 12-day fed-batch system in controlled ambr™ bioreactors. Overall, all clones presented similar growth and viability profiles, except for clone L4, which presented higher growth (Figure S1A,B). The antibody titer ranged between 0.53 and 2.3 g/L at day 12 (Octet measurement, Figure S1C). Clones were analyzed using transcriptomic and proteomic approaches at day 5 and day 10.

### 3.1. Mispairing Profile of Clones

Mispairing profiles of the clones studied in this work were reported by Tousi F. et al. [19], where the same clones were cultured under the same upstream platform and process parameters as presented in this manuscript. Clones were classified as low- or high mispairing according to their percentage of correct tsAb mass (low mispairing if percentage of correct tsAb mass ≥ 90%; and high mispairing if percentage of correct tsAb mass < 90%) [19]. More concretely, the low mispairing clones studied in this work (L1-L4) presented a % of correct tsAb mass between 90.8 and 97.2, while the high mispairing clones, H1, H2, H3, and H4, presented 48.8, 31.0, 0.0, and 0.0% correct tsAb, respectively (Figure S2A).

Besides the correct tsAb form, the different mispaired species were detected and characterized using MS, including light chain mispairing species (H1L1/H2L1 and H1L2/H2L2), heavy chain mispairing species (H2L2/H2L2) and half antibodies (H2L2 and H1L1).

Aiming at assessing if the mispaired species production resulted from bottlenecks in tsAb chain expression and/or translation, the expression levels of LC and HC transcripts and peptides in cells were analyzed. 

**Figure 1:**
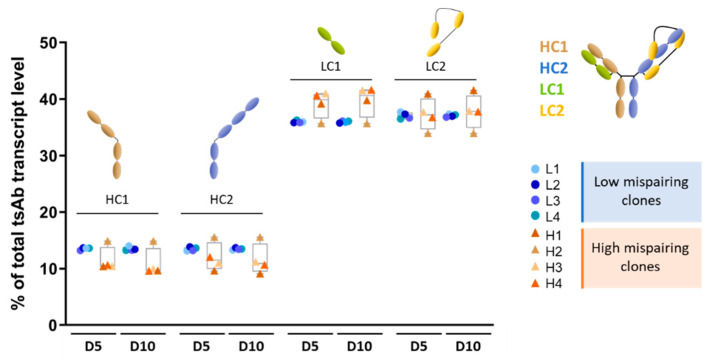
Expression of transcripts encoding different chains of tsAb were constant between low and high mispairing clones and between culture timepoints. Relative quantification (% of total tsAb chain transcript expression) of transcripts encoding for Heavy Chain 1 (HC1, brown); Heavy Chain 2 (HC2, blue); Light Chain 1 (LC1, green), and Light Chain 2 (LC2, yellow) in low and high mispairing clones at day 5 and day 10 of culture. No statistically significant differences (unpaired student *t*-test) were detected between low (L, *n* = 4) and high (H, *n* = 4) mispairing clones.

The TsAb chain gene expression showed consistency at the two time points assayed, indicating that there were no bottlenecks in transcription related to the different culture phases analyzed. A tendency (non-significant) for lower HC1 and HC2 and higher LC1 expression in high mispairing clones (orange-colored triangles) for both time points assayed was observed. Interestingly, the high mispairing clone H2 showed expression levels for HC1, HC2, and LC1 closer to the low mispairing clones (blue-colored circles) (Figure 1), probably due to the higher level of H1L2/H2L2 species that this clone presents (Figure S2B).

**Figure 2:**
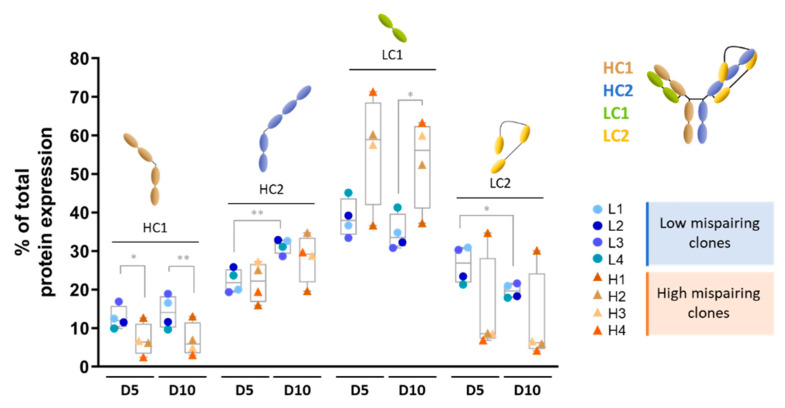
Expression of peptides encoding different chains of tsAb differed between low and high mispairing clones and by culture. Relative quantification (% of total tsAb chain protein expression) of peptides included in Heavy Chain 1 (HC1, brown); Heavy Chain 2 (HC2, blue); Light Chain 1 (LC1, green), and Light Chain 2 (LC2, yellow) in low mispairing (L, *n* = 4) and high (H, *n* = 4) mispairing clones at day 5 and day 10 of culture. * *p*-value ≤ 0.05; ** *p*-value < 0.01 (unpaired student *t*-test).

On the other hand, the TsAb chain peptide expression, obtained through SWATH-MS analysis, showed a consistently lower expression of HC1, higher expression of LC1 (with exception of clone H1), and lower expression of LC2 (with exception of clone H1) in the high mispairing clones for both time points assayed (Figure 2). These data suggest that the mispairing levels could be related to HC1, LC1, and LC2 protein expression, and that the bottlenecks in chain production occur at the translation level, rather than at transcription level. 

The peptide levels of HC2 (Figure 2) showed a higher % from day 5 to day 10, while the peptide levels of LC2 showed an opposite tendency (lower levels at day 10). 

### 3.2. Transcriptomic and Proteomic Analysis of tsAb-Producing Clones Reveals Enrichment in Key Pathways in Low vs. High Mispairing Clones

In order to identify the cellular processes involved in the level of mispairing of produced tsAbs, whole transcriptome and proteome analysis of tsAb-expressing clones was performed. Clones were analyzed at day 5 and at day 10 of culture. Gene expression was accessed using RNAseq, and proteomic profiles were evaluated through SWATH-MS.

In total, 12,043 transcripts and 1951 proteins were quantified across all samples at both time points analyzed (full list of identified transcripts and proteins in Supplementary Files S1 and S2). 

Principal component analysis (PCA) of omics datasets (Figures S3 and S4) showed that the culture time was the largest contributor (PC1) to the variation among samples, with transcript and protein expression profiles clustering according to the different culture phases, as well as clustering according to the mispairing level (PC2) for transcriptomics data. To access cellular pathways related to tsAb mispairing levels, transcript gene counts and protein peak areas were compared between the low and high mispairing clone groups. All transcripts with a *p*-value ≤ 0.05 (N = 2779 for day 5 and N = 2461 for day 10) and proteins (N = 251 for day 5 and N = 399 for day 10) were used as inputs for functional analysis (Supplementary Files S3–S6), performed using IPA software, version 101138820 (full list of identified canonical pathways and functions in Supplementary Files S7–S10). 

A large overlap was observed between the proteins and transcripts, with most proteins identified (95.4%) also being detected at transcript level (Figure S5A). Molecules quantified at both transcript and protein level with a *p*-value ≤ 0.05 (116 and 144 were assessed at day 5 and day 10, respectively) tended to present similar gene and protein expression fold change values (Figure S5B,C).

#### 3.2.1. Exponential Growth Phase (Day 5)

**Figure 3:**
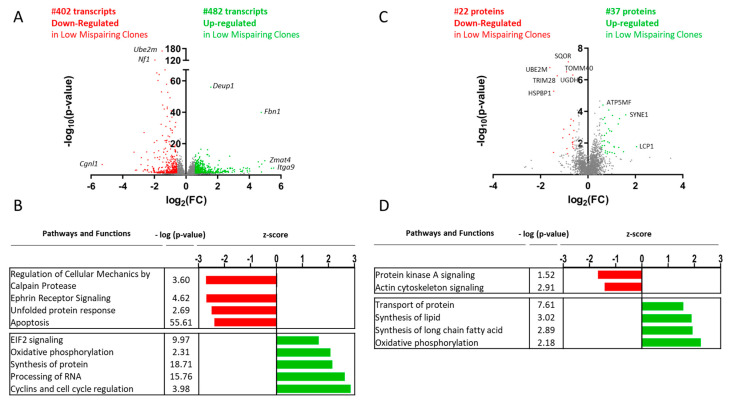
Differential gene and protein expression analysis between low and high mispairing clones in the exponential growth phase (day 5). (**A**) Volcano plot representing the transcripts sequenced and quantified through mRNA seq. This analysis enabled the identification and quantification of 12,043 transcripts, including 884 differentially expressed (log(*p*-value) ≥ 1.3) between low and high mispairing clones: 402 downregulated (log_2_(FC) ≤ −0.58, red) and 482 upregulated (log_2_(FC) ≥ 0.58, green) in low mispairing clones. (**B**) Functional analysis through IPA revealed canonical pathways and functions inhibition (negative z-score, green) and activation (positive z-score, red) predictions. (**C**) Volcano plot representing the proteins quantified by SWATH-MS. This analysis enabled the identification and quantification of 1951 proteins, including 60 differentially expressed (−log(*p*-value) ≥ 1.3) between low and high mispairing clones: 22 downregulated (log_2_(FC) ≤ −0.58, red) and 37 upregulated (log_2_(FC) ≥ 0.58, green) in low mispairing clones. (**D**) Functional analysis through IPA revealed canonical pathways and function inhibition (negative z-score, green) and activation (positive z-score, red) predictions. Only functions and pathways with a −log(*p*-value) ≥ 1.3 are represented. Genes and proteins with the highest −log(*p*-value) and highest/lowest log_2_(FC) are annotated. FC: fold change.

Functional analysis of all (*p*-value ≤ 0.05) transcripts (Supplementary File S3) and proteins (Supplementary File S4) revealed several biological functions and pathways that were activated in clones with low tsAb mispairing levels at day 5 when compared to high mispairing clones, including cell cycle regulation, synthesis of protein, oxidative phosphorylation, and EIF2 signaling. On the other hand, an unfolded protein response (UPR) presented negative z-scores in the transcriptomic analysis, suggesting the activation of this pathway in clones with higher tsAb mispairing levels (Figure 3B,D). 

A total of 884 transcripts were found to be significantly differentially expressed, including 402 transcripts downregulated (fold change ≥ 0.67, *p*-value ≤ 0.05) and 482 transcripts upregulated (fold change ≥ 1.5, *p*-value ≤ 0.05) in low mispairing clones (Figure 3A, Supplementary File S3). MA plots comparing the transcript fold change values with normalized gene count base means are depicted in Figure S6A. Regarding proteomic analysis, 60 proteins were found to be significantly differentially expressed, including 22 proteins downregulated (fold change ≥ 0.67, *p*-value ≤ 0.05) and 37 proteins upregulated (fold change ≥ 1.5, *p*-value ≤ 0.05) in low mispairing clones (Figure 3C, Supplementary File S4). 

Within the subset of differentially regulated genes and proteins, we observed an enrichment of molecules associated with cell cycle control and DNA damage response, cell stress mechanisms, phagosome formation, RNA expression, and endocytosis. Several genes and proteins associated with protein-targeted degradation via ubiquitination were also found to be differentially regulated (*Eloc*, *Fblx20, Brca1*, Brca2, and *Ube2c* genes and FBXL205 protein were found to be upregulated, while *Ube2m*, *Ube2s*, and *Spsb4* genes; ubiquitin conjugating enzyme UBE2M; and HSPBP1 protein, reported to inhibit chaperone-assisted degradation of target proteins [30]; were found to be downregulated in low tsAb mispairing clones), indicating a possible role of these pathways in the regulation of tsAb mispairing levels by cells.

Omics data also pointed towards an increased protein synthesis in low mispairing clones (z-score = 2.14), with higher gene expression levels of 10 ribosome components (Rpl19, Rpl23, Rpl30, Rps10, Rps14, Rps15, Rps20, Rps3, Rps4y1, and Rps6ka1), tRNA synthetase genes (Nars1, Tars2, Mars1, Dars1, and Grsf1), proteins (NARS1, LARS1, and TARS3), genes associated with ribonucleotide synthesis (Ak4, Dut, and Ump), genes involved in the regulation of mRNA spliceosomal cycle, important for mRNA maturation (Casc3, Cwc25, Dhx8, Edtud2, Eif4a3, Prpf19, and Rbmx2), and predicted activation of EIF2 signaling (z-score = 1.62). 

**Figure 4:**
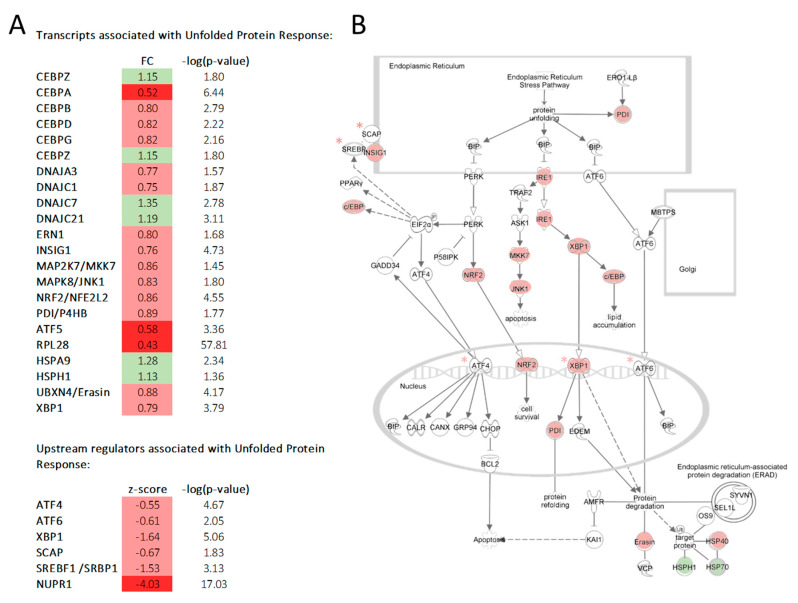
Gene expression results point to an inhibition of unfolded protein response (UPR) in low mispairing clones in the growth phase (day 5). (**A**) UPR associated transcripts are depicted as red (negative fold change (FC), dark red if FC ≤ 0.67), green (positive FC, dark green if FC ≥ 1.5. Only transcripts with −log(*p*-value) ≥ 1.3 were used as input for functional analysis. UPR upstream regulators (*) identified through IPA as inhibited (z-score ≤ −2) are also displayed. (**B**) Schematic image of UPR network was retrieved from the IPA database. Molecules shaded white were not identified in our analysis.

This observed protein synthesis activation did not seem to translate into higher endoplasmic reticulum stress in the low mispairing clones. When comparing to high mispairing clones, UPR was downregulated (by transcriptomics analysis), with clones presenting low tsAb mispairing levels, showing an overall decreased level of molecules associated with UPR mechanisms (Figure 4). In contrast to the low mispairing clones, high tsAb mispairing clones presented higher levels of endoplasmic reticulum molecular chaperones (PDI, DNAJA3, DNAJC1). Several transcription factors associated with ER stress were also predicted to be inactivated in low vs. high mispairing clones (Figure 4). Proteomic analysis also predicted XBP1 (one of the key factors in UPR signaling during ER stress) as an inhibited upstream regulator (z-score = −2.59) in these low mispairing clones.

#### 3.2.2. tsAb Production Phase (Day 10)

**Figure 5:**
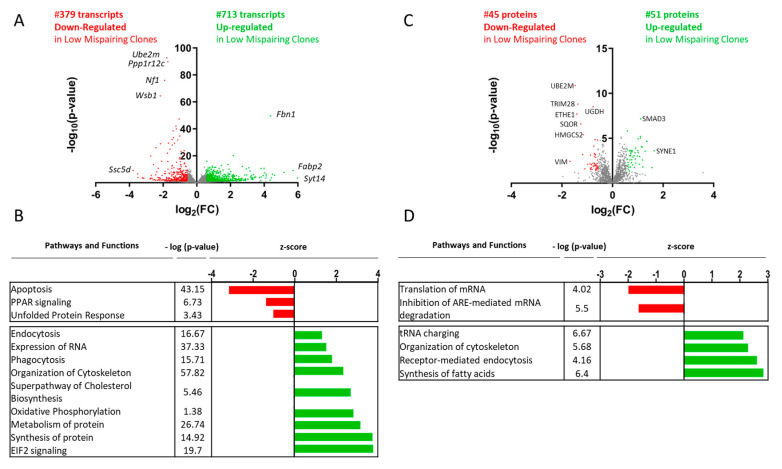
Differential gene and protein expression analysis between low and high mispairing clones in the culture production phase (day 10). (**A**) Volcano plot representing the transcripts sequenced and quantified through mRNA seq. This analysis enabled the identification and quantification of 12,043 transcripts, including 1092 differentially expressed (−log(*p*-value) ≤ 1.3) between low and high mispairing clones: 379 downregulated (log_2_(FC) ≤ −0.58, red) and 713 upregulated (log_2_(FC) ≥ 0.58, green) in low mispairing clones. (**B**) Functional analysis through IPA revealed canonical pathway and function inhibition (z-score ≤ −2, green) and activation (z-score ≥ 2, red) predictions. (**C**) Volcano plot representing the proteins quantified using SWATH-MS. This analysis enabled the identification and quantification of 1951 proteins, including 97 differentially expressed (−log(*p*-value) ≤ 1.3) between the low and high mispairing clones: 10 downregulated (log_2_(FC) ≤ −0.58, red) and 11 upregulated (log_2_(FC) ≥ 0.58, green) in low mispairing clones. (**D**) Functional analysis through IPA revealed canonical pathway and function inhibition (z-score ≤ −2, green) and activation (z-score ≥ 2, red) predictions. Only functions and pathways with −log(*p*-value) ≥ 1.3 are represented. Genes and proteins with the highest −log(*p*-value) and highest/lowest log_2_(FC) are annotated. FC: fold change.

Functional analysis of all (*p*-value ≤ 0.05) transcripts (Supplementary File S5) and proteins (Supplementary File S6) quantified at day 10 revealed that several pathways and functions, including protein synthesis, oxidative phosphorylation, and endocytosis were predicted as activated, while unfolded protein response was again identified as inactivated in clones producing low percentages of mispaired tsAb (Figure 5B,D). A total of 1092 transcripts were found to be significantly differentially expressed, including 379 transcripts downregulated (fold change ≥ 0.67, *p*-value ≤ 0.05) and 713 transcripts upregulated (fold change ≥ 1.5, *p*-value ≤ 0.05) in low mispairing clones (Figure 5A, Supplementary File S5). MA plots comparing the transcript fold change values with normalized gene count base means are depicted in Figure S6B. Regarding proteomic analysis, 97 proteins were found to be differentially expressed, including 45 downregulated (fold change ≥ 0.67, *p*-value ≤ 0.05) and 51 upregulated (fold change ≥ 1.5, *p*-value ≤ 0.05) in low mispairing clones (Figure 5C, Supplementary File S6). Differentially expressed molecules were highly associated with cell cycle regulation (79 transcripts and 11 proteins associated with cell cycle regulation were identified as upregulated), endocytosis, protein degradation, stress response, and RNA transcription.

The gene and protein expression results also indicate the activation of endocytosis in clones with lower mispairing levels (z-score of 2.91 and 2.51 for transcript and protein expression analysis), with 85 genes (including 46 genes with FC ≥ 1.5) and 12 proteins (including 5 with FC ≥ 1.5) associated with endocytosis mechanisms presenting higher expression in the low mispairing clone group. 

Similarly to what was observed at day 5, at day 10, the functional analysis also pointed towards decreased UPR and activated tRNA charging (z-score = 2.12, with DARS1, IARS1, NARS1, KARS1, YARS1, FARSB, and TARS1 tRNA ligases presenting higher protein expression values in low mispairing clones). The tRNA ligase genes *Eprs1*, *Iars2*, *Lars2*, and *Nars1* also presented the same expression pattern. 

#### 3.2.3. RNA-Seq Validation

**Figure 6:**
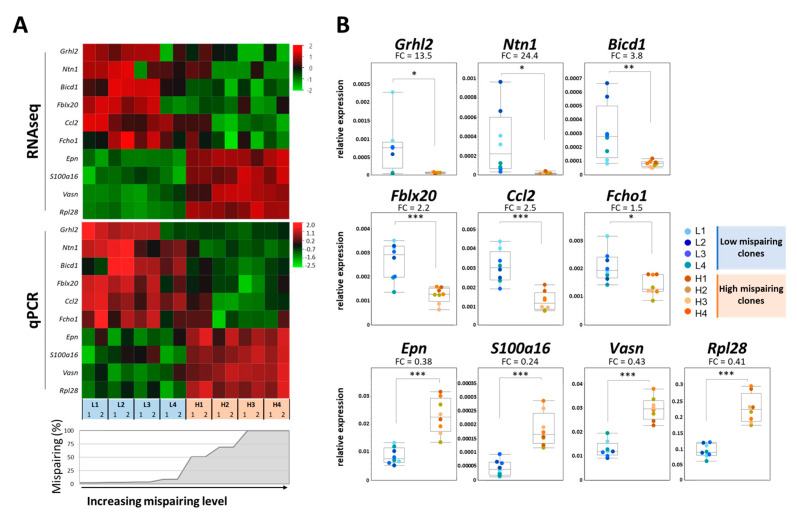
Expression profile of *Grhl2*, *Ntn1*, *Bicd1*, *Chst8*, *Fblx20*, *Ccl2*, *Fcho1*, *Epn*, *S100a16*, *Vasn*, and *Rpl28* transcripts and validation using qPCR. Expression profile in the exponential growth phase of the culture of the selected transcripts was accessed using RNAseq and validated through qPCR. Expression values were normalized to the housekeeping gene β-actin. (**A**) Heatmap (transformed log2 z-score) highlighting RNAseq and qPCR differential expression gene patterns between high (H) and low (L) mispairing groups. (**B**) qPCR quantification of transcripts in low mispairing (blue circles) and high mispairing (orange circles) clones. * *p*-value ≤ 0.05; ** *p*-value ≤ 0.01; *** *p*-value ≤ 0.001: (unpaired student *t*-test), (FC = fold change).

In order to validate the RNA-seq data, several differentially expressed transcripts were selected based on the differential expression between high and low mispairing clones and quantified across all day-5 samples using real-time quantitative PCR. Similar results were obtained with both methodologies, with all genes validated as significantly regulated (*p*-value ≤ 0.05, FC ≤ 0.67 or ≥1.5), indicating the robustness and reliability of the RNA-seq results (Figure 6). 

## 4. Discussion

Strategies to optimize recombinant protein production in CHO hosts are mainly focused on transgene and vector engineering [31,32] and the optimization of extracellular bioprocess parameters (pH, temperature, medium design, fed-batch vs. perfusion culture methods, etc.) [19,33,34,35]. Host cellular pathways and mechanisms have also been investigated to improve monospecific and MsAb titer [36,37,38,39,40], glycosylation [41,42], product clipping [43], and aggregation profiles [34,39]. However, the host cellular processes linked to MsAb mispairing remain largely uncharacterized. 

**Figure 7:**
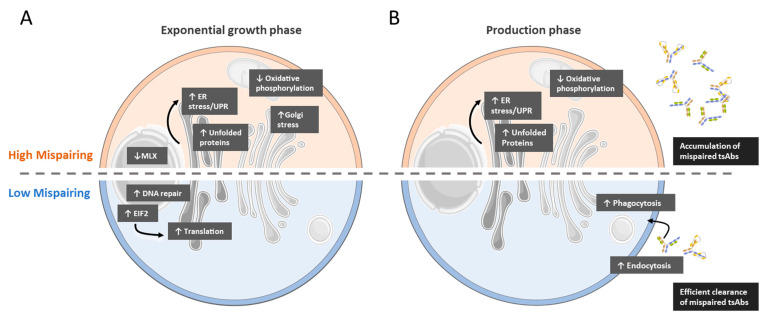
Omics analysis revealed the main differences between low and high mispairing clones. Pathways found to be activated (↑) and inactivated (↓) in both phases of culture and in both groups of clones are depicted. (**A**) In the exponential growth phase (day 5), gene expression and proteomic analysis indicated increased EIF2 signaling and mRNA translation in low mispairing clones, while in high mispairing clones, endoplasmic reticulum unfolded protein response and Golgi stress were activated. (**B**) In the stationary growth/antibody production phase (day 10), gene expression and proteomic analysis indicated higher levels of endocytosis and phagocytosis in low mispairing clones, while high mispairing clones retained the molecular signature of increased ER stress.

To gain further insights into specific intracellular events and pathways that may play a role in determining MsAb chain mispairing, we investigated the gene and protein expression profiles of different CHO clones producing the same tsAb with different mispairing levels. Functional analysis of omics data suggested that clones presenting high mispairing profiles present bottlenecks in mRNA translation regulation and increased ER stress and UPR, potentially due to reduced efficiency in protein folding and tsAb chain assembly. Endocytosis and phagocytosis are also activated in low mispairing clones, suggesting that these cells have more efficient clearance mechanisms to deal with misfolded species (Figure 7).

Production of a mAb starts with mRNA transcription of the different chains. Expression vectors are usually tailored to favor the expression of excess LC over HC, as this improves antibody secretion and quality, due to the specificities of mAb assembly kinetics [44,45,46,47]. However, for more complex formats, such as MsAbs, this general guideline may not apply, as excess LC may pair with non-cognate HC, creating mispaired species (LC1-HC2 and LC2-HC1) [37,44]. In this work, higher LC vs. HC transcript levels were detected in all clones analyzed; however, no significant differences between clones with different mispairing levels were observed. 

Upon transcription, regulation of mRNA translation was reflected differently in the levels of detected tsAb chain peptides between the low and high mispairing clones, reinforcing the important role of mRNA translation regulation. Unlike transcript levels, significant differences between HC and LC polypeptide levels were detected between the high and low mispairing clones. Namely, there was a consistently higher expression of LC1 and lower expression of LC2 and HC1 polypeptides in high mispairing clones, except for clone H1. Interestingly, the high mispairing clones presented higher percentages of H2L2 species. A lower percentage of HC1 polypeptide in the high mispairing clones might indicate a correlation with the amount of available HC1 peptide and the produced MsAb species (richer in HC2). The same trend, however, was not observed for LC1 peptide. Although there were higher levels of detected LC1 peptides in the high mispairing clones, this did not translate into higher levels of LC1 rich species. Accumulation of LC1 chain in the ER of high mispairing clones might have induced chain processing bottlenecks and ER stress, as seen for another MsAb (bispecific), where, after investigating the individual and pairwise chain expression of two LCs and two HCs, the authors found that one of the LCs was not secretion competent, accumulating in the ER and causing high mispairing levels [44]. 

Several modulators of mRNA translation, including EIF2 signaling, were found to be differentially modulated in low vs. high mispairing clones at day 5. At day 10, low mispairing clones presented activated tRNA charging and activated protein synthesis functions (with higher expression of several translation initiation factors and ribosomal proteins in low mispairing clones). Other modulators of mRNA translation presented higher levels of protein expression in low vs. high mispairing clones, including KHSRP (involved in mRNA stabilization [48,49]), CSTF3 (involved in the polyadenylation and 3’-end cleavage processing required for the maturation of pre-mRNA into functional mRNAs [50]), and ABCF1 and gene *Abcf1* (required for Cap and IRES mediated mRNA translation initiation [51]. Of the six translation initiation factor protein subunits identified, five presented higher expression in low mispairing vs. high mispairing clones (EIF2B4, EIF3C, EIF3F, EIF3K, and EIF3L). Moreover, components of the cleavage and polyadenylation specificity factor complex (*Cpsf2*, *Cpsf3,* and *Cpsf4*), *Celf2*, also involved in regulation of pre-mRNA maturation, and regulator of transcription elongation factor *Cdk12* also presented higher expression levels in the low mispairing clones.

Despite this evidence that mRNA translation is activated in low mispairing clones, proteomic analysis yielded negative z-score for translation of mRNA at day 10. When investigating more closely the proteins associated with this function (*n* = 13), we can see that the negative z-score was mainly supported by the upregulation of DHFR (FC = 1.69), FXR1 (FC = 1.18), IGF2BP2 (FC = 1.18), and DDX3X (FC = 1.13). DHFR inhibition by methotrexate (MTX) was the selection method used for the development of the clones involved in this study [19]. Therefore, overexpression of DHFR might be a result of other factors, such as productivity levels, other than mRNA translation regulation. FXFR1 has been implicated in the regulation of cytokine TNF translation [52], while IGF2B2 promotes the stability and transient storage of its target mRNAs [53]. On the other hand, DDX3X has been reported to regulate overall mRNA translation, including promotion of translation initiation, repressing translation in cells under stress and in cap-dependent translation [54,55,56]. Neither DHFR, FXFR1, nor IGF2B2 seem to have an impact on the inhibition of overall cellular mRNA translation, which might hamper the correct interpretation of the regulation level of this pathway.

Overall, the functional data analysis showed that protein synthesis was activated in low mispairing clones, with higher expression of tRNA synthetases, higher expression levels of translation initiation and elongation factors, and ribosome components at both time points analyzed.

Different MsAb polypeptide chains are synthesized separately by ribosomes and translocated from the cytoplasm to the ER, where post-translational modifications, folding, maturation, and chain assembly take place. Accumulation of unfolded or misfolded proteins leads to the activation of the UPR pathway, an adaptative mechanism that seeks to overcome ER stress by increasing the capacity of cells to fold proteins through upregulation of chaperone expression, attenuation of mRNA translation, increasing ER volume through stimulating production of membrane lipids, and degradation of unfolded proteins via the ER-associated protein degradation pathway (ERAD) [57]. Indeed, several CHO cell engineering strategies targeting the over-expression of genes involved in ER stress response have shown benefits for improving monospecific and MsAb productivity [36,37,38,39,58], antibody clipping [43], and aggregation [34,39].

In this study, omics expression data were indicative of higher ER stress in high mispairing clones in both culture phases analyzed, with upregulation of several key genes and proteins associated with UPR, including several chaperones, while other molecules involved in ERAD. XBPI, ATF4, ATF6, and NUPR1 transcription factors, which act as master regulators or ER stress, were also predicted to be inactivated in the low vs. high mispairing clones. Enzymes associated with the crosstalk between ER and cholesterol biosynthesis, such as *Srebf1* and *Scap* transcription factors [59], were also predicted to be inhibited in low mispairing clones. At D10, this tendency was inversed, with *Srebf1*, *Srebf2,* and *Scap* transcription factors predicted to be activated, as well as other transcripts and proteins involved in cholesterol synthesis and regulated by *Srebf2* [60,61,62]. 

Besides higher ER stress, the lower expression of *Mlx* transcript, a key transcriptional repressor of the Golgi stress response [63], also points towards an activated Golgi stress response in clones with high mispairing at day 5. Several genes and proteins associated with protein traffic through the ER–Golgi axis were also differentially expressed between high and low mispairing clones, including the downregulation of adaptor protein genes *Ap4s1* and *Ap1g2*, and ADP-ribosylation factor gene *Arf3*. Several of the genes found with lower expression in low mispairing clones are associated with retrograde protein transport, including *Arcn1*, *Scfd1*, and the sortins *Snx1* and *Snx27*, indicating that high mispairing clones have activation of retrograde protein transport. Retrograde targeting to ER has been associated with receptor trafficking, antigen cross-presentation by dendritic cells, and also protein degradation through the ERAD machinery [64,65]. 

Neutrophil cells express receptors for the constant region of IgG immunoglobulins (FcγRs) that recognize and internalize antigen-bounded and free antibodies [66]. Endocytosis of extracellular antibody species has not yet been described for producing cell lines, and, although alterations in glycosylation [67,68] and Fab region structure [69] have been shown to modulate antibody-FcγRs binding affinity, it is still unclear how the detection of misfolded domains would be detected in the extracellular milieu. Nevertheless, activation of endocytosis, phagocytosis, *Fcgr2*, and *Fcr4* (member of FcγRs family) transcripts in low mispairing clones suggests that low mispairing clones were better equipped for extracellular detection, uptake, and targeted degradation of mispaired tsAB species at day 10.

Eleven transcripts that better distinguished low mispairing and high mispairing clones (*Grhl2*, *Ntn1*, *Bicd1*, *Fblx20*, *Ccl2*, *Fcho1*, *Epn*, *S100a16*, *Vasn*, and *Rpl28*) were selected to validate the RNAseq transcript expression data through qPCR. From the expression profiling data, the quantification of these genes’ expression has the potential to be used as a biomarker panel tool to identity high mispairing levels early in cell line and bioprocess development, reducing the timelines and costs in MsAb product development. 

To our knowledge, this is the first study to utilize omic quantitative data to investigate the intracellular mechanisms underlying mispairing in MsAb formats. While the small cohort of samples used in this study and the unbalanced distribution of productivity levels of the groups analyzed here could have impacted the statistical power of the findings, the combination of robust transcriptomics and proteomics quantitative analytical technologies enabled us to unveil several clues about the relation between mispairing profiles and key intracellular pathways. High mispairing clones present bottlenecks in terms of mRNA translation, chain processing, as well as mispaired antibody degradation. Defects in MsAb folding and assembly seem to lead to an activation of UPR and Golgi stress responses. Taken together, this work provides new insights and raises new questions regarding MsAb mispairing quality control by the cell host. The data generated provide a basis for future studies on the selection of targets for host cell engineering, aiming at generating hosts that will produce higher quality MsAb products. Moreover, several transcripts and proteins that correlate with tsAb mispairing levels can also be exploited for the development of a biomarker panel that could be applied as an additional tool for the early screening and selection of clones with more suitable product profiles, favoring hosts with improved MsAb quality and reducing MsAb development and manufacturing process time and costs.

## Figures and Tables

**Table 1:** Sequences of primers used in the PCR analysis.

Transcript	Forward	Reverse
β-actin	5′-ATGACGATATCGCTGCGCTC-3′	5′-ATGGCTACGTACATGGCTGG-3′
Ntn1	5′-ACTGTGACTCCTATTGCAAGGC-3′	5′-TTGTC CGCTTTCAGGATGTGGA-3′
Fcho1	5′-CTGCTGTCCAAGAACCTCTTCG-3′	5′-AAAGGGGATGGGCTGGATGTGA-3′
Eps	5′-GGCTCAATGACCACGGCAAGAA-3′	5′-ACTGGAAGTCCTTCAGCGTCTG-3′
Rpl28	5′-CCACCATCAACAAGAATGCACGG-3′	5′-GTGCGCTTTCTCTTCACCACCA-3′
Fbxl20	5′-CAGTAACTGGCAACGGATAGACC-3′	5′-CCTACTCCAAGACACCCACGAA-3′
Bicd1	5′-CATCAAGGAAAGGAGAATCC-3′	5′-GTTTGTGACTCCTGGAGGTTGG-3′
Ccl2	5′-GCTACAAGAGGATCACCAGCAG-3′	5′-GTCTGGACCCATTCCTTCTTGG-3′
Grhl2	5′-GGACGTGAATGAAGAGGCAAAG-3′	5′-TTGACAGTACGCTCTGTGGATG-3′
S100a16	5′-TGTTTCCAAGCACAGCCTGGTC-3′	5′-TGGTTGGCATCCAGGTTCTGGA-3′
Vasn	5′-CCAGCG TCCACCTGCCTGAATG-3′	5′-CTTGCCTCACAGGACTCTCACA-3′

## Data Availability

The relevant data to understand and replicate the results reported in this work are within the manuscript and its supporting information files. Raw transcriptomic and proteomic data, data containing antibody targets, antibody sequence, and culture medium used (owned by Sanofi) cannot be shared publicly due to legal restrictions.

## Supplementary Materials

The following supporting information can be downloaded at: https://www.mdpi.com/article/10.3390/biomedicines11112890/s1, Figure S1: Growth and titer profile of CHO clones fed-batch cultures; Figure S2: Mispairing profile of the different clones; Figure S3: Principal component analysis (PCA) of gene expression results of all samples, including technical replicates; Figure S4: Principal component analysis (PCA) of protein expression results of all samples, including technical replicates; Figure S5: Molecules commonly identified in transcriptomic and proteomic analysis; Figure S6: MA plot displaying the log fold-change compared with base mean expression generated by DESeq2 transcriptomics datasets. File S1: Full list of quantified transcripts in the RNASeq analysis; File S2: Full list of quantified proteins in the SWATH-MS analysis; File S3: List of genes identified in low vs. high mispairing clones in exponential growth phase (D5) with *p*-value ≤ 0.05; File S4: List of proteins identified in low vs. high mispairing clones in exponential growth phase (D5) with *p*-value ≤ 0.05; File S5: List of genes identified in low vs. high mispairing clones in tsAb production phase (D10) *p*-value ≤ 0.05; File S6: List of proteins identified in low vs. high mispairing clones in tsAb production phase (D10) with *p*-value ≤ 0.05; File S7: Full list of canonical pathways, functions and diseases from IPA functional analysis of transcriptomics data D5 low vs. high mispairing clones; File S8: Full list of canonical pathways, functions and diseases from IPA functional analysis of transcriptomics data D10 Low vs. High mispairing clones; File S9: Full list of canonical pathways, functions and diseases from IPA functional analysis of proteomics data D5 low vs. high mispairing clones; File S10: Full list of canonical pathways, functions and diseases from IPA functional analysis of proteomics data D10 low vs. high mispairing clones.
