# Supplementary material for: Identification of Mispairing Omic Signatures in Chinese Hamster Ovary (CHO) Cells Producing a Tri-Specific Antibody
Source: Biomedicines. 2023 Oct 25;11(11):2890. doi: 10.3390/biomedicines11112890 (PMC10669571; doi:10.3390/biomedicines11112890)
Supplement: Supplementary file 1 [file biomedicines-11-02890-s001.zip › Supplementary Figures.pdf]

Supplementary Figures

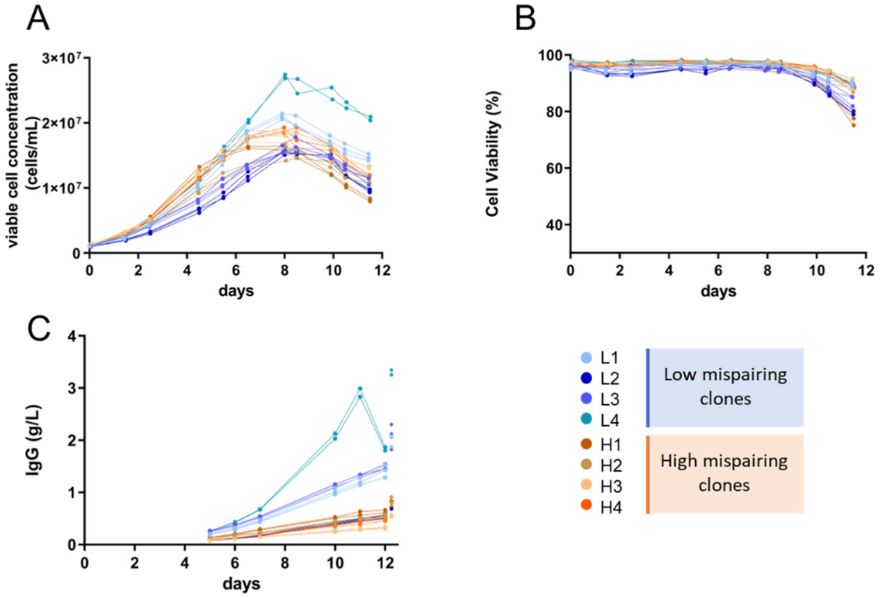

Figure S1. Growth and titer profile of CHO clones fed-batch cultures. CHO clones, with low (blue) and high (orange) tsAb production mispairing profiles were expanded in fed-batch conditions. A) Viable cell density; B) Cell Viability; C) Titer

A

Clone ID	% correct tsAb mass	Classification
L1	97.2	Low Mispairing
L2	96.7	
L3	96.0	
L4	90.8	
H1	48.8	High Mispairing
H2	31.0	
H3	0.0	
H4	0.0	

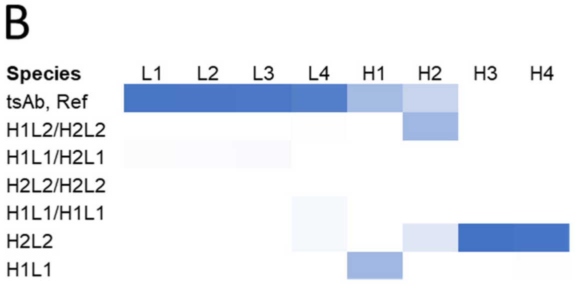

Figure S2. Mispairing profile of the different clones. A) Clones were classified as Low or High mispairing depending on their relative quantification of correct tsAb mass (*low mispairing if percentage of correct tsAb mass ≥ 90%; and high mispairing if percentage of correct tsAb mass < 90%*). B) Types and distribution of tsAb species. Data was retrieved from Tousi et al 2020[19].

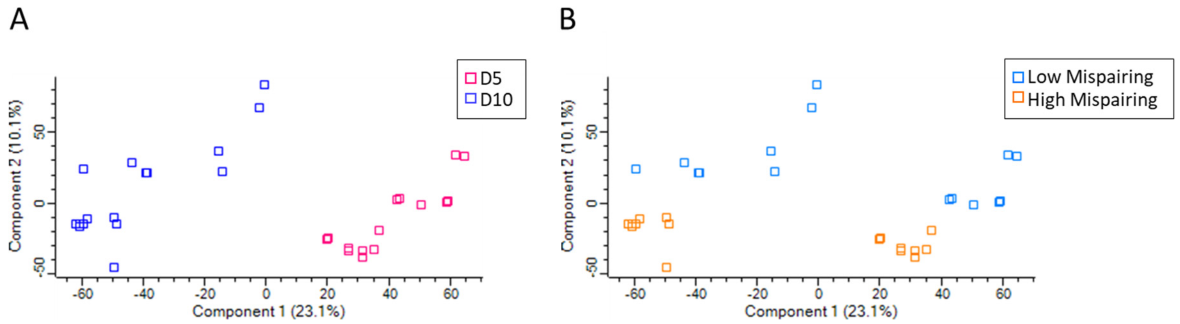

Figure S3. Principal Component Analysis (PCA) of gene expression results of all samples, including technical replicates (PCA settings: Unsupervised; Cutoff method: Benjamin-Hochberg). Scores for PC1 (23.1%) vs PC2 (10.1%) are displayed. A) Samples are grouped according to day (Pink: day 5; Dark Blue: day 10). B) Samples are grouped by mispairing level (Light Blue: low mispairing; Orange: high mispairing).

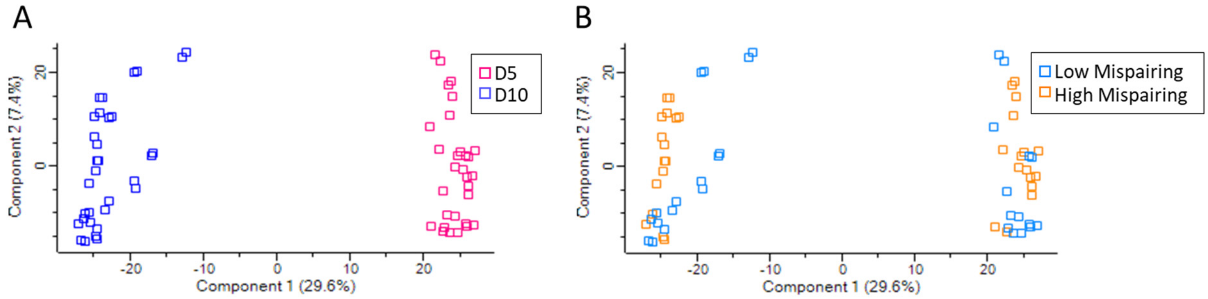

Figure S4. Principal Component Analysis (PCA) of protein expression results of all samples, including technical replicates (PCA settings: Unsupervised; Cut-off method: Benjamin-Hochberg). Scores for PC1 (29.6%) vs PC2 (7.4%) are displayed. A) Samples are grouped according to day (Pink: day 5; Dark Blue: day 10). B) Samples are not clustered by mispairing level (Light Blue: low mispairing; Orange: high mispairing).

A

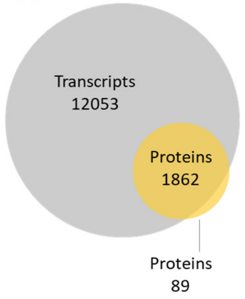

B

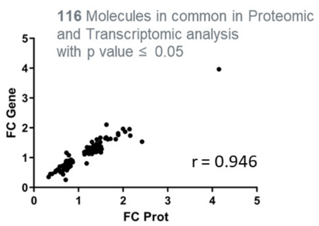

	Fold Change	
	Transcript	Protein
Nf1	0.26	0.71
Ube2m	0.35	0.33
Rab34	0.44	0.66
Poldip2	0.44	0.66
Hspbp1	0.45	0.37
Trim28	0.46	0.41
Ppp2r1a	0.51	0.60
Ethe1	0.52	0.48
Bmp1	0.56	0.64
Hpcal1	0.56	0.49
Hk2	0.57	0.54
Mov10	0.58	0.74
Sqor	0.60	0.56
Tomm40	0.60	0.53
Tle3	0.63	0.68
Idh1	0.66	0.77
Septin4	0.67	0.78
Septin6	0.69	0.74
Ptgfrn	0.70	0.71
Akr1b8	0.70	0.58
Hexa	0.70	0.80
Txndc5	0.70	0.64
P3h4	0.71	0.82
Brcc3	0.73	0.69

	Fold Change	
	Transcript	Protein
Gsta3	0.73	0.65
Kcnab2	0.74	0.78
Lgals3	0.75	0.69
Pdia4	0.77	0.77
Uap1	0.77	0.78
Atxn10	0.78	0.70
Pdxdc1	0.79	0.75
Serpinh1	0.80	0.71
Ephx1	0.80	0.80
Iqgap2	0.81	1.18
Esd	0.82	0.81
Actr3	0.82	0.83
Fkbp9	0.83	0.72
Gnb1	0.83	0.75
Sec23a	0.84	0.75
Gnai2	0.85	0.87
Grb2	0.86	0.82
Capns1	0.86	0.72
Pgrmc1	0.87	0.84
Tpm4	0.87	0.81
Sec31a	0.88	0.85
Arf4	0.88	0.79
Prkar1a	0.89	0.72
P4hb	0.89	0.74

	Fold Change	
	Transcript	Protein
Ywhab	0.89	0.77
Appl1	0.89	0.85
Rer1	0.90	0.86
Psmb4	0.90	0.82
Anxa2	0.93	0.83
Rbbp7	1.08	0.78
Puf60	1.11	1.13
Eif3d	1.13	1.30
Slk	1.13	1.23
Eif3a	1.15	1.26
Eef1g	1.15	1.26
Cpsf3	1.15	1.23
Usp47	1.16	1.16
Khsrp	1.16	1.27
Syncrip	1.16	1.21
U2af1	1.17	0.73
Stat3	1.18	1.50
Rpa1	1.18	1.29
Pafah1b3	1.18	1.20
Cct5	1.18	1.13
Babam2	1.19	1.39
Tst	1.19	1.30
Npm1	1.20	1.50
Tcp1	1.20	1.15

	Fold Change	
	Transcript	Protein
Iqgap1	1.20	1.26
Dhx30	1.21	1.31
Acsl4	1.21	1.43
Nmt1	1.21	1.48
Tubgcp2	1.23	1.33
Slc25a12	1.26	1.40
Ltn1	1.26	1.45
Fuca1	1.27	1.23
Lars1	1.28	1.48
Sh3gl1	1.28	1.51
Nsun2	1.28	1.41
Lpl	1.29	1.41
Mcmbp	1.29	1.52
Dis3	1.29	1.32
Nudc	1.31	1.21
Prmt5	1.31	1.45
Trap1	1.33	1.41
Gclm	1.34	1.24
Fh	1.35	1.19
Arhgap18	1.37	1.50
Cav1	1.37	1.39
Nup85	1.38	1.38
Nup107	1.38	1.41
Elac2	1.40	1.33

	Fold Change	
	Transcript	Protein
Prim1	1.45	1.43
Itgb5	1.46	1.40
Eftud2	1.52	1.49
Kpnb1	1.53	1.30
Txnl1	1.54	2.42
Rcc2	1.61	1.41
Impa2	1.61	1.66
Smad3	1.62	1.84
Top2a	1.63	1.72
Rab2a	1.65	1.60
Atp5f1a	1.67	1.49
Lman1	1.68	1.62
Med1	1.74	2.16
Afg3l2	1.76	1.89
Napg	1.81	1.89
Fbxl20	1.87	2.05
NARS1	1.95	2.14
Isoc1	1.97	2.00
Me2	2.11	1.63
Lcp1	3.97	4.15

C

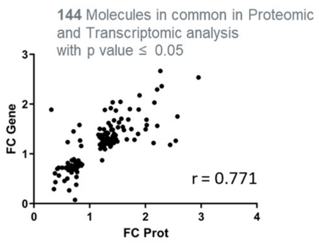

	Fold Change	
	Transcript	Protein
Ssc5d	0.07	0.74
Nf1	0.27	0.63
Ube2m	0.29	0.36
Poldip2	0.38	0.76
Trim28	0.43	0.39
Hspbp1	0.46	0.55
Tomm40	0.50	0.60
U2af2	0.54	0.76
Hmgcs2	0.56	0.45
Ethe1	0.61	0.38
Slc27a4	0.63	0.67
Hexa	0.63	0.77
Dpp7	0.67	0.76
Htatip2	0.67	0.62
Slc25a24	0.69	0.72
Aldh1a1	0.69	0.61
P3h4	0.69	0.67
Sqor	0.70	0.42
Hpcal1	0.72	0.57
Luc7l	0.72	0.75
P2rx4	0.72	0.51
Ptgfrn	0.72	0.44
Ctsb	0.74	0.60
Dnaja1	0.74	0.79
Ctsd	0.75	0.76
Acot7	0.76	0.78
Itpr3	0.76	0.69
Acss2	0.76	0.79
Mical1	0.76	0.85

	Fold Change	
	Transcript	Protein
Hibadh	0.77	0.67
Blmh	0.78	0.80
Abr	0.78	0.86
Eif5	0.79	0.86
Arl8a	0.79	0.76
Blvrb	0.80	0.83
Cndp2	0.80	0.77
Dhrs1	0.80	0.79
Man2a1	0.80	0.80
Psmd14	0.81	0.83
Lrpap1	0.82	0.61
Hadh	0.83	0.78
Gfpt1	0.83	0.74
Psme4	0.87	0.80
Gnb1	0.87	0.68
Cct3	0.87	1.22
Prdx5	0.89	0.72
Eif3c	1.09	1.27
Eif3l	1.10	1.19
Usp47	1.13	1.22
Kpna3	1.14	1.22
Cct2	1.15	1.37
Kif2a	1.15	1.26
Usp46	1.16	0.85
Cct6a	1.17	1.23
Ank3	1.17	1.24
Rab5c	1.17	1.38
Ppm1g	1.18	2.44
Eif3f	1.19	1.18

	Fold Change	
	Transcript	Protein
Slc25a12	1.20	1.20
Csnk2a2	1.20	1.21
Snd1	1.20	1.26
Rala	1.20	1.32
Ppp2r1b	1.22	2.15
Psme3	1.22	1.32
Aplp2	1.22	1.43
Cpsf3	1.22	1.36
Fh	1.22	1.29
Tjp1	1.22	1.43
Rps15	1.24	0.61
Plod1	1.24	1.25
Cct8	1.25	1.23
Rsu1	1.25	1.50
Tmpo	1.25	1.39
Gnai3	1.26	1.36
Glg1	1.26	2.54
Tcp1	1.27	1.35
Ak2	1.27	0.83
Mtap	1.27	1.41
Diaph1	1.28	1.35
Tst	1.28	1.38
Maoa	1.28	1.24
Stat3	1.28	1.80
Dnm2	1.29	1.33
Rcc2	1.29	1.67
Kpnb1	1.29	1.47
Dock1	1.30	1.30
Jade1	1.31	1.43

	Fold Change	
	Transcript	Protein
Mmut	1.31	1.34
Pdia6	1.31	1.32
Myh10	1.32	1.20
Ubash3b	1.32	1.23
Xpo5	1.34	1.31
Efr3a	1.34	1.18
Eef1g	1.34	1.23
Igf2bp2	1.35	1.18
Abcf1	1.35	1.18
Nup155	1.35	1.20
Pxdn	1.35	1.41
Acly	1.37	1.47
Ehd3	1.38	1.20
Dlst	1.38	1.16
Cad	1.39	1.43
Prmt5	1.40	1.27
Txnl1	1.40	1.87
Hmgcs1	1.40	1.44
Dis3	1.42	1.29
Cnot11	1.42	1.33
Ipo5	1.43	1.31
Ehd1	1.45	0.71
Msh2	1.48	1.61
Actn1	1.48	1.39
Txnrd1	1.49	1.42
Atp6v1h	1.49	1.26
Plod2	1.49	2.00
Plxna1	1.50	1.75
Gstm3	1.50	1.18

	Fold Change	
	Transcript	Protein
Lss	1.53	1.51
Cobll1	1.54	1.59
Sqle	1.56	1.38
Acat2	1.56	1.38
Ncam1	1.56	2.21
NARS1	1.58	2.02
Arpc1b	1.58	0.82
Anxa11	1.62	1.34
Impa2	1.63	1.53
Csnk1a1	1.67	1.44
Cav1	1.69	2.02
Dhfr	1.69	1.69
Rab2a	1.71	1.57
Fbxl20	1.75	2.57
Tubb6	1.87	1.47
Eef2	1.88	1.27
Vim	1.89	0.31
Arhgap18	1.89	1.88
Atp5f1a	1.90	1.72
Lman1	1.92	1.38
Smad3	2.03	2.16
Lpl	2.04	1.41
Dab2	2.05	1.55
P3h2	2.18	1.89
Itgb5	2.29	2.21
Stat5a	2.36	2.29
Lcp1	2.54	2.95
Isoc1	2.67	2.27

Figure S5. Molecules commonly identified in transcriptomic and proteomic analysis. A) Most of the identified proteins were also identified at the transcript level. From analysed molecules with p-value ≥0.05, B) 47 were identified at both transcript and protein level at exponential growth phase (day 5), with Pearson correlation coefficient r=0.909and C) 51 were identified at both transcript and protein level at tsAb production phase (day 10), with Pearson correlation coefficient r=0.630. With some exceptions, molecules present similar expression trends when detected as transcript or proteins.

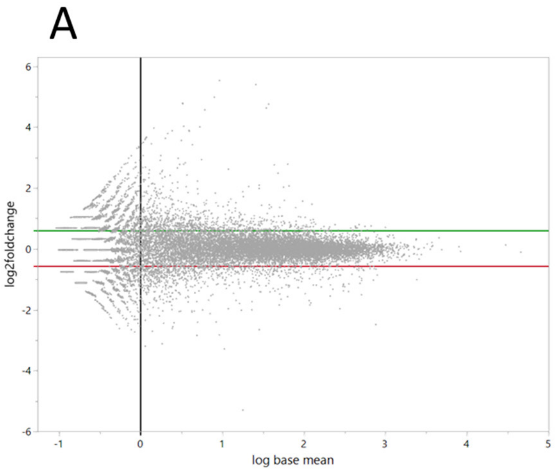

Figure S6. MA plot displaying the log fold-change compared with base mean expression generated by DESeq2 transcriptomics data sets. A) Day 5 – exponential phase B) Day 10- production phase. Default log fold-change thresholds of 0.58 (FC≥1.5) and -0.58 (FC≤0.67), and log base mean threshold of 0 (base mean≥1) are represented in the graph by green, red and black lines respectively.
